# Comparison of endoscopic thyroidectomy via the oral vestibule approach and the areola approach for papillary thyroid carcinoma

**DOI:** 10.1186/s12893-024-02413-3

**Published:** 2024-04-27

**Authors:** Yingying Liu, Fusheng Lin, Wei Yan, Ende Lin, Penghao Kuang, Xiaoquan Hong, Yizhuo Lu, Guoyang Wu, Lianghui Li

**Affiliations:** 1 Department of Anesthesiology, Zhongshan Hospital of Xiamen University, Xiamen, China; 2 Department of General Surgery, Zhongshan Hospital of Xiamen University, 201-209 Hu Bin Nan Road, Xiamen, China

**Keywords:** Endoscopic thyroidectomy, Oral vestibular approach, Areola approach, Papillary thyroid carcinoma, Surgical completeness

## Abstract

**Background:**

The endoscopic thyroidectomy areola approach (ETAA) has been widely applied for papillary thyroid carcinoma (PTC), but leaves scars and is not truly minimally invasive. The oral vestibular approach (ETOVA) leaves no scars and is even more minimally invasive. However, there have been few comparative studies of ETAA and ETOVA for PTC. The purpose of our research was to compare two PTC treatment methods in terms of feasibility, safety, efficacy, and cosmetic results.

**Methods:**

A total of 129 patients with PTC underwent thyroidectomy combined with central lymph node dissection by the same surgeon. Among them, 79 patients underwent the ETOVA, and the others underwent the ETAA. We compared the two groups in terms of operative outcomes, postoperative complications, and cosmetic results.

**Results:**

No significant differences were found in the clinical characteristics between the ETOVA and ETAA groups. There were no significant differences in the number of removed lymph nodes (*P = 0.279*) or the number of positive lymph nodes (*P = 0.569*), but the ETOVA group had a higher number of removed lymph nodes. There was also no significant difference in blood loss volume(*P = 0.180*), postoperative drainage volume (*P = 0.063*), length of hospital stay (*P = 0.182*), transient RLN injury rate (*P = 1.000*), permanent RLN injury rate (*P = 1.000*), or recurrence rate (*P = 1.000*). The ETOVA was a longer operation than the ETAA was (*P < 0.01*). The ETOVA group had less pain (VAS 1: *P* < 0.01, VAS 3: *P = 0.001*), less neck discomfort (1 month after surgery: *P = 0.009*, 3 months after surgery: *P = 0.033*), and better cosmetic results (*P = 0.001*).

**Conclusions:**

The ETOVA is not inferior to the ETAA in terms of safety and curability of PTC and is advantageous in terms of central lymph node dissection, minimal invasiveness, and cosmetic results.

**Trial registration:**

This study was approved by the Ethics Committee of Zhongshan Hospital of Xiamen University (2017 V1.0). No funding was received.

**Supplementary Information:**

The online version contains supplementary material available at 10.1186/s12893-024-02413-3.

## Background

Thyroid cancer is the most common endocrine cancer, with a particular predominance of women [1, 2]. Papillary thyroid carcinoma (PTC) is the most common subtype, accounting for approximately 85% of thyroid carcinomas [3]. Although open thyroidectomy (OT) is a routine treatment method for PTC, it inevitably leaves surgical scars on the neck, affecting aesthetic outcomes. With increasing concerns about surgical trauma and cosmetic outcomes, especially among young women, endoscopic thyroidectomy (ET) is increasingly performed for the treatment of PTC. Since ET was first introduced by Hüscher in 1997 [4], various endoscopic thyroid surgeries, including axillary [5], axillary breast [6], submental [7], and areola approaches (ETAA) [8], have been introduced by many surgeons. In the past, comparative studies of ET and OT have shown that ET was not inferior to OT in terms of effectiveness and safety and was superior to OT in terms of aesthetic outcomes [9–11]. Nevertheless, these endoscopic surgeries still require incisions in the areola and/or axillary fold, and because of the need for extensive dissection, they are not truly minimally invasive. On the other hand, the oral vestibular approach (ETOVA) introduced by Wang [12] and Anuwong [13] leaves no scars and is even more minimally invasive. However, there have been few comparative studies of ET, such as ETOVA, and ETAA. The purpose of our research was to compare two PTC treatment methods in terms of feasibility, safety, efficacy, and cosmetic results.

## Materials and methods

### Patient information

From August 2017 to December 2019, 79 patients with PTC underwent the ETOVA at Zhongshan Hospital of Xiamen University, while another 50 patients with PTC underwent the ETAA during the same period. The indications for ETOVA or ETAA in our study were as follows: PTC diagnosed by fine-needle aspiration biopsy, tumor diameter less than 3 cm, no central lymph node metastasis, no cervical lymph node metastasis, and no distant organ metastasis. The contraindications were as follows: history of neck surgery or ablative therapy, oral malformation or local infection, severe thyroiditis, hyperthyroidism, large tumors located in the upper pole of the thyroid gland, extrathyroidal invasion or other organ or systemic complications that prevent the patient from tolerating surgical trauma or general anesthesia. All patients were operated on by the same surgeon. All enrolled patients signed informed consent forms, and this study was approved by the ethics committee of our institute.

We analyzed the data of the ETOVA group and the ETAA group and compared them in terms of operative outcomes (operative time, blood loss volume, drainage amount, length of hospital stay, number of dissected lymph nodes, and number of positive lymph nodes), postoperative complications [recurrent laryngeal nerve (RLN) palsy, superior laryngeal nerve (SLN) injury, hypocalcemia, pain, neck discomfort, and recurrence], and cosmetic results.

Laryngoscopy was routinely performed to assess vocal cord function before and after surgery. Given the difficulty of diagnosing SLN dysfunction through clinical manifestations or endoscopy, detection of the SLN by IONM at the end of the operation was used for postoperative evaluation of SLN function. The occurrence of a cricothyroid twitch after stimulation of the SLN at the end of the operation indicated good nerve function. Moreover, the SLN was determined to have impaired functionality. The diagnostic criteria for hypoparathyroidism were a serum parathyroid hormone (PTH) concentration less than 15 ng/L, a serum calcium concentration less than 2.0 mmol/L, and the need for calcium supplementation to alleviate symptoms. On the first morning and the third morning after surgery, all the enrolled patients, under the guidance of a professional nurse, used the visual analog scale (VAS) to score their pain. All the patients were evaluated for cosmetic outcomes using a scoring system (1, extreme; 2, fair; 3, normal; 4, not at all) 6 months after the operation [9]. The cosmetic results were evaluated by one doctor who was unaware of the surgical methods. All the patients underwent B-ultrasonography once one year after the operation. If there was suspicion of recurrence or metastasis, fine needle aspiration (FNA) and CT were routinely performed for definitive diagnosis.

### Surgical approach

#### ETOVA operative technique

**Fig. 1:**
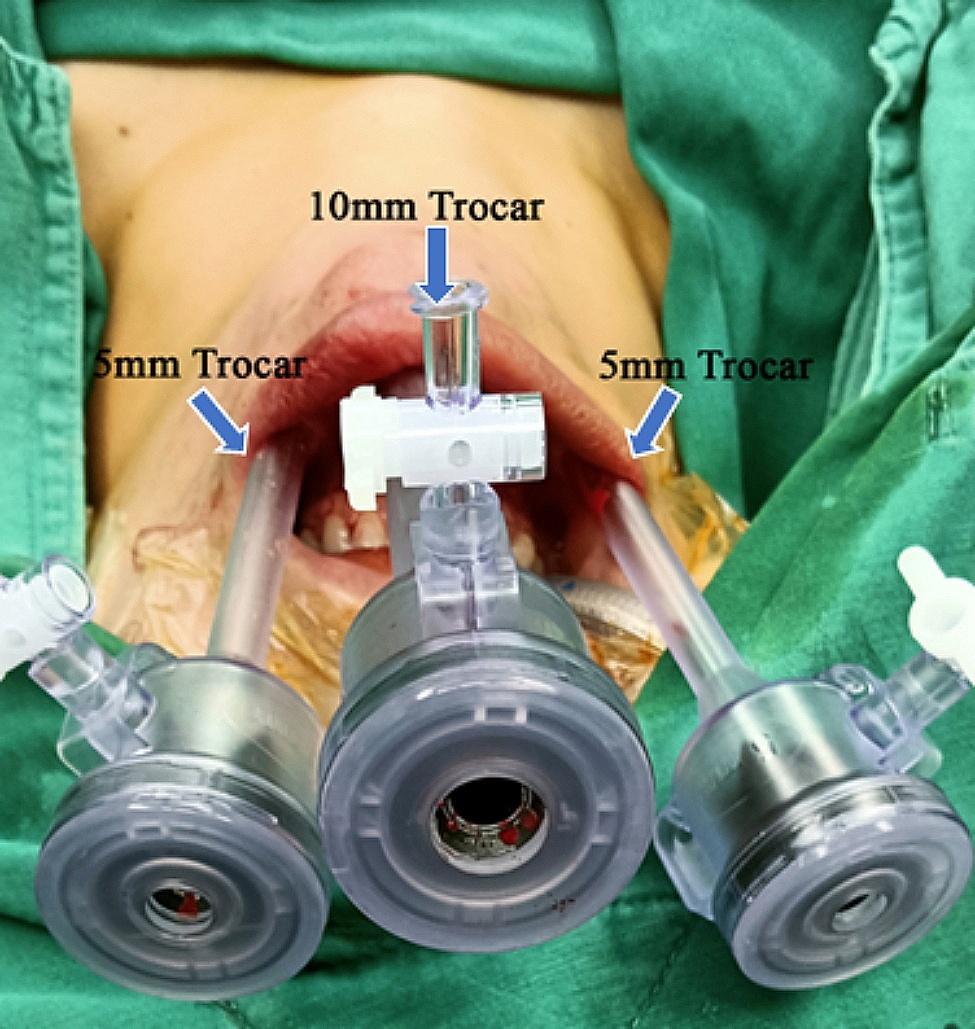
Location of the trocar in the ETOVA

**Fig. 2:**
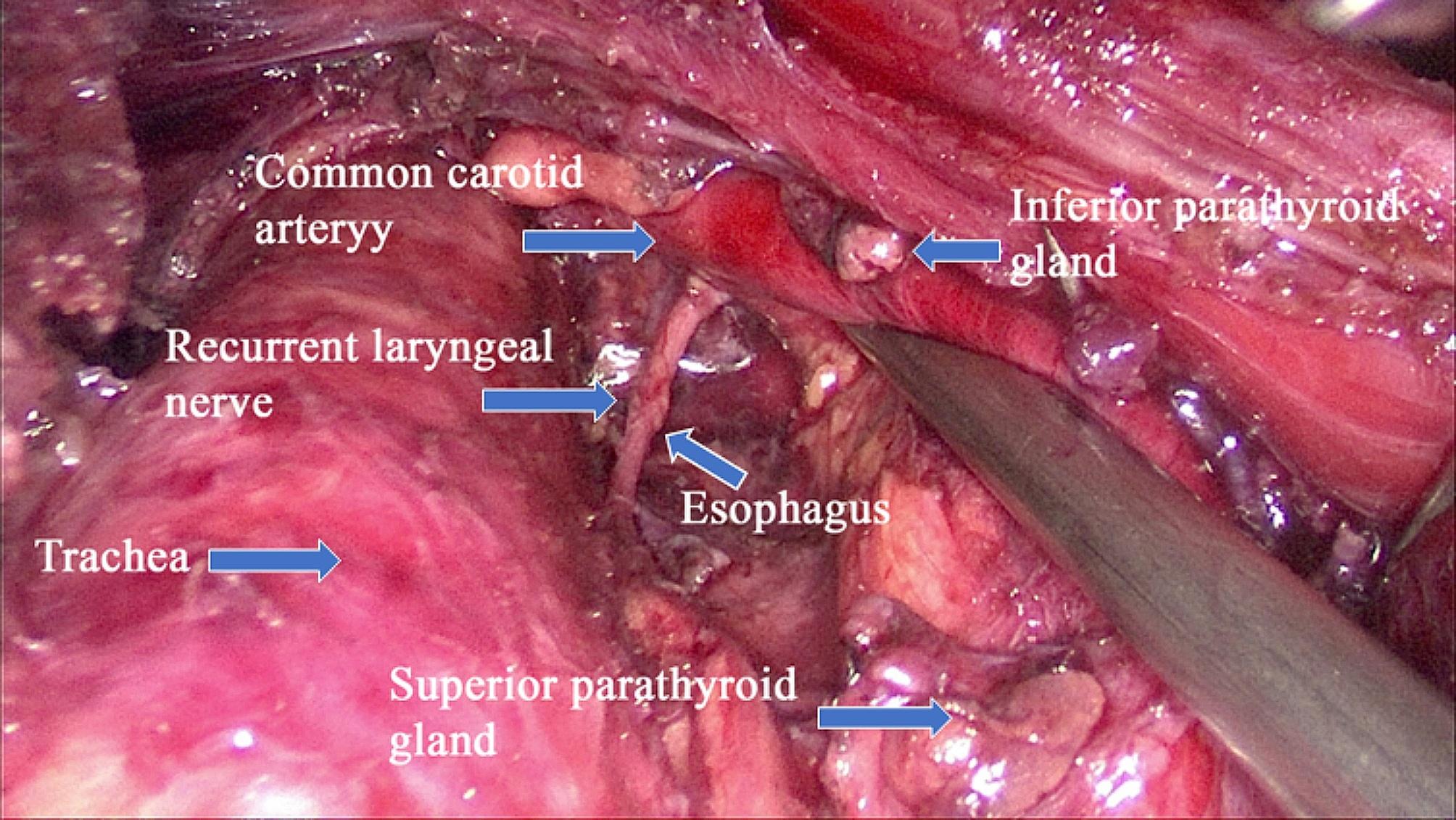
Surgical field after central lymph node dissection via the ETOVA

The patient received general anesthetics through oral endotracheal intubation using an NIM 3.0 EMG tube (Medtronic, Minneapolis, MN, USA) and was placed in a supine position with the neck slightly extended. The surgeon stood at the patient’s head with the camera assistant on his left side, and the video monitor was placed at the patient’s feet. After oral disinfection with iodine solution, a transverse mucosal incision was made in front of the lower lip frenum. With blunt dissection performed in the subplatysmal plane, a 10 mm trocar was introduced, and the space was insufflated with carbon dioxide (CO_2_) at a pressure of 4 mmHg. The other two 5 mm incisions were made close to the corner of the mouth, and two 5 mm ports were then inserted into the same space (Fig. 1). The surgical space was expanded along the deep surface of the platysma, reaching downward to the suprasternal notch and laterally to the sternocleidomastoid muscle. The linea alba was separated from the thyroid cartilage to the sternal notch, and then the isthmus of the thyroid was divided along the surface of the trachea. After traction of the strap muscle with a needle-shaped hook, the outer edge of the thyroid lobe was detached, and the middle thyroid vein was removed. The superior pole of the thyroid lobe was gently pulled downward to expose the sternothyroid–laryngeal triangle. Then, blunt separation of the superior pole of the thyroid was performed under direct vision, followed by ligation of the superior thyroid artery on the thyroid capsule. During this process, intraoperative neural monitoring (IONM) was used to monitor the SLN in the sternothyroid–laryngeal triangle. The RLN was then identified and dissected along the trachea–esophageal groove. An ultrasonic scalpel was used to cut the inferior thyroid artery that was tightly adhered to the inferior pole of the thyroid, thereby avoiding causing damage to the RLN, and the thyroid lobe was completely removed. Then, the lymph node in the central area was separated from the external side along the surface of the common carotid artery, from the internal side along the surface of the trachea, downward to the level of the innominate artery, and backward to the esophagus and prevertebral fascia (Fig. 2). During the operation, the ultrasonic blade was kept as far away as possible from the trachea and nerve, and the signal of the RLN was checked via IONM. The “uncapping” method is commonly used to manage the superior pole of the thyroid, with preservation of the posterior blood supply of the upper pole a high priority. Due to its relatively fixed anatomical location, the superior parathyroid could be easily identified and retained in situ. The inferior parathyroid should be retained in situ as much as possible during lymph node clearance in the central area. Once the gland cannot be retained in situ, autotransplantation into the sternocleidomastoid muscle or subcutaneous tissue in the upper arm is performed. If total thyroidectomy and bilateral central lymph node dissection were needed, the opposite lobe and central lymph node were removed in the same manner. The specimen was bagged and completely removed through the observed trocar. After careful hemostasis, the wound was repeatedly rinsed with sterile distilled water to avoid tumor implantation. A negative pressure drainage tube was introduced through the supraclavicular fossa, and then the incision of the oral mucosa was sutured with absorbable sutures (Detail in Supplementary Fig. 1).

#### ETAA operative technique

**Fig. 3:**
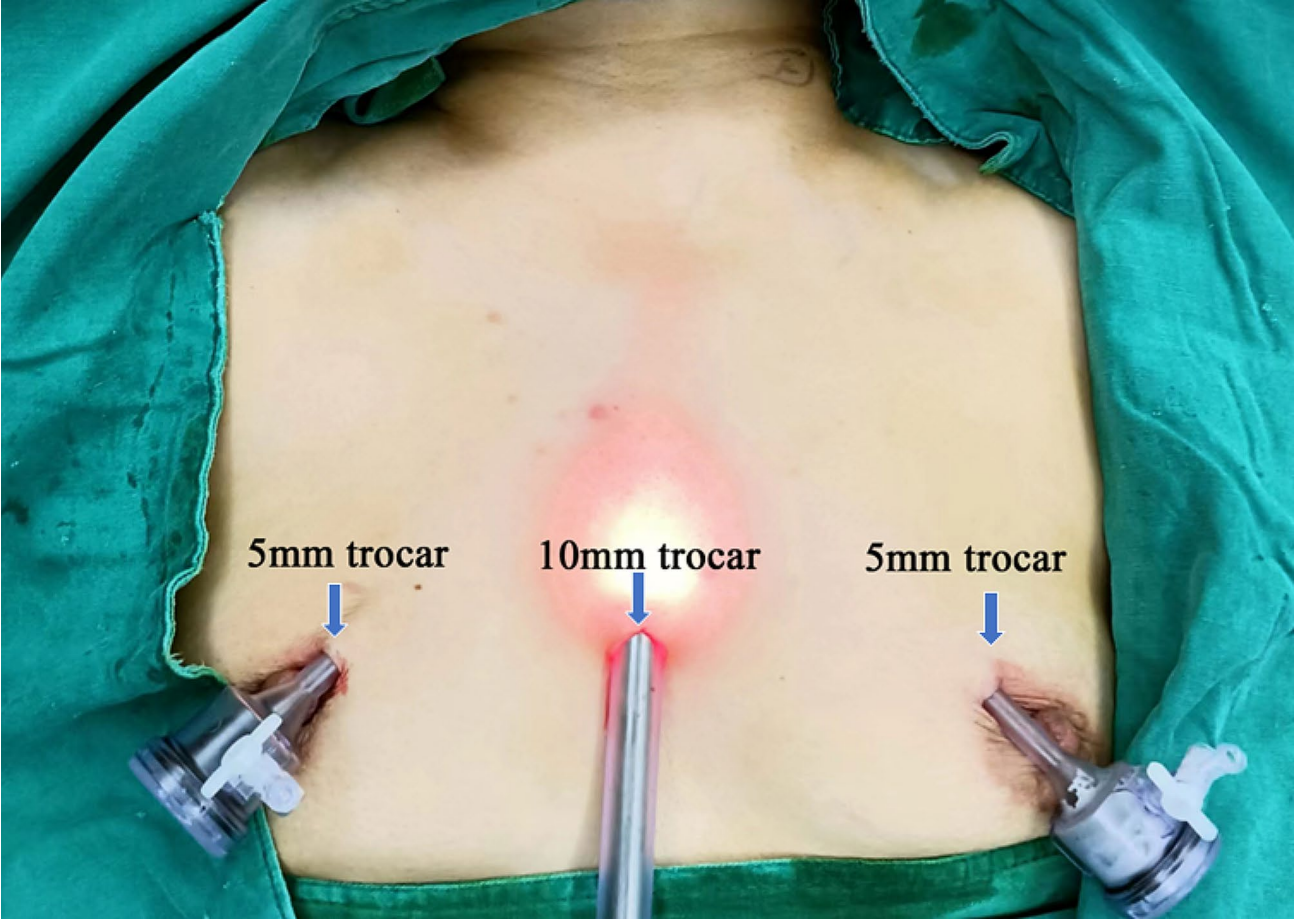
Location of the trocar in the ETAA

**Fig. 4:**
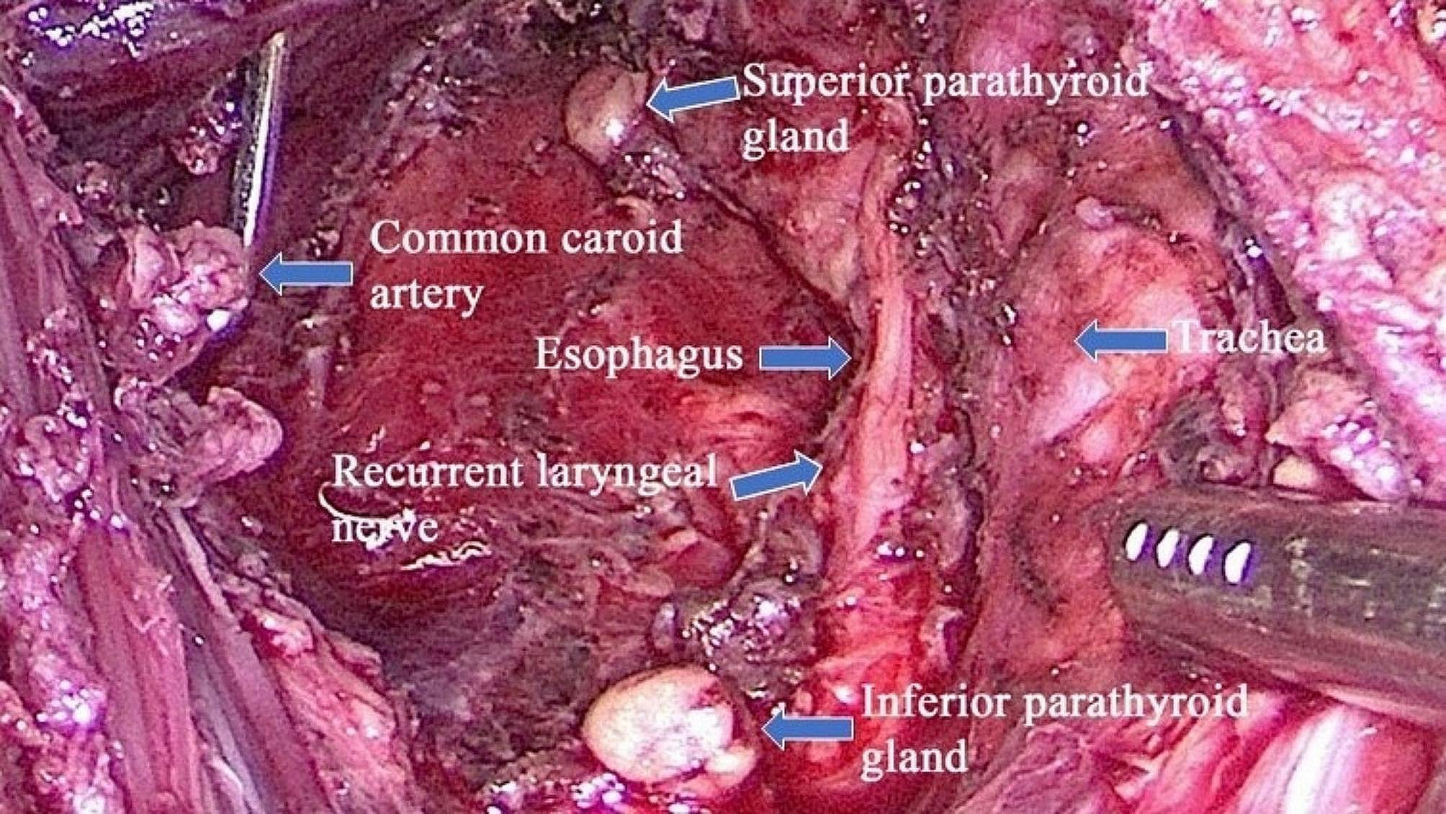
Surgical field after central lymph node dissection via the ETAA

General anesthesia was induced through oral endotracheal intubation using a NIM 3.0 EMG tube (Medtronic, Minneapolis, MN, USA). The patient was placed in the supine position with the legs apart and the neck slightly extended. The television monitor was positioned at the patient’s head, and the surgeon stood between the patient’s legs with the assistant on one side of the patient. After a 10 mm incision was made at the midpoint of the two nipples, a 10 mm trocar was inserted through this incision as an observation hole. CO_2_ was insufflated through the observation hole at a pressure of 8 mmHg, and then two 5 mm trocars were placed at the edges of the left and right areolas on the left side at 10–11:00 and on the right side at 1–2:00 (Fig. 3). The operation space was expanded to the surface of the pectoralis major, upward to the thyroid cartilage level, and on both sides to the outer edge of the sternocleidomastoid muscle. The linea alba was separated from the sternal notch to the thyroid cartilage, and then the isthmus of the thyroid was divided along the surface of the trachea. After traction of the strap muscle with a needle-shaped hook, the outer edge of the thyroid lobe was detached, and the middle thyroid vein was removed. With traction of the thyroid lobe upward, coagulation was observed close to the lower pole of the thyroid lobe in the inferior thyroid artery. Then, the RLN was exposed in the tracheal-esophageal groove, and the inferior parathyroid was identified and preserved as much as possible. The dorsal side of the thyroid lobe was separated above the entrance of the RLN into the larynx. Eventually, coagulation was observed in the superior thyroid artery, and the superior parathyroid was recognized and preserved. The methods used for SLN exploration and superior thyroid artery dissection were the same as those used for ETOVA. After the lobe was dissected completely, ipsilateral central compartment node dissection was routinely performed. The degree of central lymph node clearance was the same as that in ETOVA, but the order of clearance was reversed, starting from the leg and ending at the head (Fig. 4). The inferior parathyroid should be preserved in situ as much as possible; otherwise, autotransplantation should be performed. Eventually, the specimen was bagged and retrieved through the observation trocar. If total thyroidectomy and bilateral central lymph node dissection were needed, the opposite lobe and central lymph node were removed in the same manner. During the operation, the ultrasonic blade was kept as far away as possible from the trachea and nerve, and the RLN signal was checked via IONM. After a surgical drain was placed through an operation trocar, the strap muscles were approximated (Detail in Supplementary Fig. 2).

### Statistical analysis

All the statistical data were analyzed by SPSS (SPSS version 18.0, Chicago, IL). All the data are expressed as the means ± SDs, proportions, or numbers. Continuous data were compared using t tests, and categorical data were analyzed using chi-square tests. A P value < 0.05 was considered to indicate statistical significance.

## Results

The clinical characteristics of the two groups are shown in Tables 1 and 2. Seventy-nine patients underwent the ETOVA, and 50 patients underwent the ETAA. Thyroidectomy (lobectomy or total thyroidectomy) and central compartment node dissection (unilateral or bilateral node dissection) were performed for both groups. No patients in either group were converted to open surgery. The pathological characteristics of both groups, such as the median age of the patients (36.63 ± 8.11 years vs. male-to-female ratio (17: 62 vs. 11:39, *P* = 0.949), tumor size (7.84 ± 4.24 mm vs. 9.40 ± 7.51 mm, *P* = 0.187), incidence of extrathyroidal extension (14.49% vs. 13.63%, *P* = 0.912), and incidence of capsular invasion (14.49% vs. 16.27%, *P* = 0.826) were not significantly different between the two groups. No significant difference was found between the two groups in terms of the number of removed lymph nodes (6.93 ± 5.35 vs. 5.92 ± 4.86, *P* = 0.279) or the number of positive lymph nodes (0.84 ± 1.75 vs. 1.04 ± 2.00, *P* = 0.569), but the ETOVA group had a higher number of removed lymph nodes. Although the ETOVA was a longer operation (162.53 ± 35.10 min vs. 122.82 ± 34.14 min, *P* < 0.01), no significant difference in the duration of total thyroidectomy (204.50 ± 35.31 min vs. 155.60 ± 55.64 min, *P* = 0.056), blood loss volume (27.84 ± 11.39 ml vs. 25.00 ± 12.16 ml, *P* = 0.180), postoperative drainage volume (168.35 ± 77.25 ml vs. 144.60 ± 57.07 ml, *P* = 0.063) or length of hospital stay (4.18 ± 0.97 days vs. 3.96 ± 0.90 days, *P* = 0.182) was observed between the two groups. Two patients in the ETOVA group and 1 patient in the ETAA group experienced transient RLN injury and recovered after 2–3 months of conservative treatment (2.5% vs. 2.0%, *P* = 1.000). Two patients in the ETOVA group and 1 patient in the ETAA group developed permanent RLN injury (2.5% vs. 2.0%, *P* = 1.000). No complications, such as superior laryngeal nerve injury, hypoparathyroidism, subcutaneous emphysema, pneumomediastinum, tracheal injury, esophageal injury, mental nerve injury, or wound infection, were found postoperatively in either group. Pain in the ETOVA group was milder than that in the ETAA group (VAS 1: *P* < 0.01, VAS 3: *P* = 0.001). Compared with those in the ETAA group, fewer patients in the ETOVA group experienced neck discomfort (1 month after the operation: *P* = 0.009, 3 months after the operation: *P* = 0.033). The postoperative cosmetic scores were 1.07 ± 0.26 in the ETOVA group and 1.34 ± 0.47 in the ETAA group (*P = 0.001*), which indicated that the cosmetic effect of the ETOVA was better than that of the ETAA. The mean follow-up period was 50.45 ± 6.73 months in the ETOVA group and 52.60 ± 7.32 months in the ETAA group (*P = 0.091*). One patient in the ETOVA group experienced tumor recurrence in the other thyroid lobe 21 months later, while another patient in the ETAA group experienced tumor recurrence in the other thyroid lobe 17 months later (1.2% vs. 2.0%, *P* = 1.000).

**Table 1:** Clinical characteristics of the patients (*n* = 129)

Variable	ETOVA (n=79)	ETAA(n=50)	P value	
Age (y)	36.63 ± 8.11	37.06 ± 8.23	0.773	t = 0.290
Sex				
Male	17	11	0.949	χ2 = 0.004
Female	62	39		
Surgical extent				
lobectomy	69	45	0.646	χ2 = 0.211
Total thyroidectomy	10	5		
Diameter of largest tumor (mm)	7.84 ± 4.24	9.40 ± 7.51	0.187	t = 1.332
Extra thyroidal extension				
Absent	69	44	0.912	χ2 = 0.012
Present	10	6		
Capsule invasion				
Absent	69	43	0.826	χ2 = 0.048
Present	10	7		
I/II/III/IV	75/2/2/0	48/1/1/0		
Number of dissected lymph nodes	6.93 ± 5.35	5.92 ± 4.86	0.279	t = 1.087
Number of positive lymph nodes	0.84 ± 1.75	1.04 ± 2.00	0.569	t = 0.572
Operative time (min)	162.53 ± 35.10	122.82 ± 34.14	0.000	t = 6.326
Lobectomy	156.44 ± 30.84	119.17 ± 29.68	0.000	t = 6.399
Total thyroidectomy	204.50 ± 35.31	155.60 ± 55.64	0.056	t = 2.095
Blood loss during surgery (ml)	27.84 ± 11.39	25.00 ± 12.16	0.180	t = 1.347
Lobectomy	26.52 ± 10.95	23.77 ± 11.73	0.206	t = 1.271
Total thyroidectomy	37.00 ± 10.59	36.00 ± 11.40	0.869	t = 0.168
Amount of drainage (ml)	168.35 ± 77.25	144.60 ± 57.07	0.063	t = 1.874
Lobectomy	162.46 ± 77.06	139.66 ± 56.28	0.090	t = 1.708
Total thyroidectomy	209.00 ± 68.91	189.00 ± 48.27	0.574	t = 0.577
Hospital stay after operation (day)	4.18 ± 0.97	3.96 ± 0.90	0.182	t = 1.342
Lobectomy	4.15 ± 1.02	3.86 ± 0.84	0.113	t = 1.598
Total thyroidectomy	4.40 ± 0.51	4.80 ± 1.09	0.344	t = 0.981

**Table 2:** Postoperative outcomes of the patients (*n* = 129)

Variable	ETOVA (n=79)	ETAA(n=50)	p	
Transient laryngeal nerve injury	2 (2.5%)	1 (2.0%)	1.000	
Permanent laryngeal nerve injury	2 (2.5%)	1 (2.0%)	1.000	
Superior laryngeal nerve injury	0	0		
Hypoparathyroidism	0	0		
Other complications	0	0		
VAS pain score				
Postoperative day 1	3.30 ± 0.42	4.29 ± 0.41	0.000	t = 13.113
Postoperative day 3	2.35 ± 0.38	3.79 ± 3.66	0.001	t = 3.458
Number of patients with neck discomfort				
1 month postoperatively	7	13	0.009	χ2 = 6.866
3 months postoperatively	2	7	0.033	χ2 = 4.564
Cosmesis	1.07 ± 0.26	1.34 ± 0.47	0.001	t = 3.567
Recurrence	1 (1.2%)	1 (2.0%)	1.000	
Follow-up period (months)	50.45 ± 6.73	52.60 ± 7.32	0.091	t = 1.702

## Discussion

The prognosis of surgical treatment for PTC is generally good; therefore, both operators and patients pay more attention to the trauma and aesthetic outcomes of surgery. With the development of relevant equipment and technology, endoscopic surgery has also been developed for the treatment of thyroid diseases. Hüscher performed the first ET in 1997 [4]. Thereafter, various endoscopic operative methods, including the axillary approach [5], axillary breast approach [6], submental approach [7], and ETAA [8], were introduced by many surgeons. In the past, numerous studies have focused on comparisons of ET and OT and have confirmed that ET has aesthetic advantages and is not inferior to OT in terms of safety and efficacy. Unlike endoscopic thyroid surgeries, endoscopic thyroidectomy via an oral approach is a type of NOTES (natural orifice transluminal endoscopic surgery) [14]. In 2011, Wilhelm reported endoscopic transoral thyroidectomy through the floor of the mouth [15]. Subsequently, an improved endoscopic transoral thyroidectomy known as the ETOVA has been gradually popularized and applied [12, 13, 16]. Zheng et al. believed that the ETOVA was equivalent to OT in terms of effectiveness and safety in treating PTC [5]. To date, there have been few comparative studies of ETOVA and other endoscopic methods for PTC. The purpose of our research was to compare the ETOVA and the ETAA for PTC in terms of feasibility, safety, efficacy, and cosmetic results.

Although Wilhelm reported the use of transoral thyroidectomy through the floor of the mouth early [15], this method has rarely been used due to damage to the oral floor tissue. However, the ETOVA has been highly recommended because it can avoid this drawback [12, 13]. However, one of the main complications of the ETOVA is numbness in the lower lip and chin, which is caused by the operating trocar being too close to the branches of the mental nerve, resulting in damage to the mental nerve. Therefore, we have made improvements by placing the operating trocar as close to the corner of the mouth as possible, effectively preventing damage to the mental nerve. Our results also showed that there were no cases of numbness in the lower lip or chin.

The lower boundary of lymph node dissection in the central region was the level of the brachiocephalic trunk. Due to the obstruction of the sternum and clavicle, the visual field for lymph node clearance was limited in the ETAA group. Thus, sufficient dissection of the cervical lateral lymph node is relatively difficult in patients who undergo the ETAA due to inadequate exposure [10]. Compared with the ETAA, the ETOVA is advantageous in that the field of vision during central lymph node dissection is unobstructed, allowing the same extent of low central node dissection as that of open surgery [17, 18]. Although no significant difference was found in the number of central lymph nodes removed between the two groups in our research, the number of lymph nodes removed in the ETOVA group was higher. Therefore, we believe that compared with the ETAA, it is more convenient and thorough to clear the central lymph nodes via the ETOVA.

The duration of the ETOVA was much longer than that of the ETAA in our research. This may be attributed to three reasons. First, operators have more difficulty in managing the affected thyroid lobe due to the narrow space in the ETOVA, while a larger operating space could be gained in the ETAA, allowing the surgeon to more easily manage the thyroid lobe. Second, the surgeon needed to overcome their previous visual habits because the endoscope in the ETOVA provided a cranio-caudal view compared with traditional methods of endoscopic surgery [11, 12, 19]. Third, even if the surgeon has experience in open surgery and endoscopic surgery, the learning curve for the ETOVA is longer [19, 20]. Our results also showed that there was no significant difference in the duration of total thyroidectomy between the ETOVA and ETAA groups. We believe that although it was difficult to process the gland via the ETOVA due to limited space, it was easier and more time-efficient to clear the central lymph nodes via the ETOVA due to the lack of visual obstruction, which to some extent compensated for the gap in work time between the two methods.

Injury to the RLN is the main complication and focus of attention for patients undergoing thyroidectomy. The incidence of transient and permanent RLN injury after open thyroidectomy ranges from 2.11 to 11.8% and 0.2–5.9%, respectively [13]. The magnification effect of the endoscope makes the RLN easier to identify [10], and IONM technology may be used to locate and identify the RLN, helping to reduce the incidence of RLN injury [21]. IONM and visual recognition of the RLN during thyroid surgery have gained widespread acceptance as the gold standard [22, 23]. Because of the same surgical equipment used for the ETAA and the ETOVA in our study, the rates of RLN injury were similar. In this study, we found 2 cases of RLN paralysis in the ETAA group, one of which was a temporary injury (resolved 1 month after surgery) and one of which was a permanent injury. We also found 3 cases of RLN paralysis in the ETOVA group, two of which were temporary injuries (resolved 1.5 months after surgery), and 1 of which was a permanent injury. The injured RLN was intact, but the nerve monitoring signal weakened or disappeared. We determined that the nerve damage was caused by thermal injury caused by the use of an ultrasonic scalpel when clearing the central lymph node. Many surgeons have summarized their experience in using an ultrasonic scalpel to avoid causing thermal injury to the nerve—particularly keeping the head of the knife head away from the nerve, maintaining the knife at a distance of at least 3 mm from the nerve, and working as quickly as possible [9, 24]. Our results showed that it was difficult to maintain the ultrasonic knife at a safe distance when separating the central lymph tissue close to the RLN, which was most likely the cause of thermal injury to the nerve. Therefore, in such a case, it may be safer to use scissors instead of an ultrasound knife. We believe that in both the ETAA and the ETOVA, thermal injury to the RLN caused by an ultrasonic scalpel is a serious problem that needs to be treated. Notably, the experience and meticulous skill of surgeons play an irreplaceable role in reducing the incidence of thermal damage caused by ultrasonic scalpels [25]. The postoperative function of the recurrent laryngeal nerve is currently evaluated via laryngoscopy. In recent years, transcutaneous laryngeal ultrasonography has been applied in clinical practice as an inexpensive and noninvasive detection method [26]. The sensitivity and specificity of detecting vocal cord function are comparable to those of laryngoscopy. Therefore, transcutaneous laryngeal ultrasonography is expected to become a reliable alternative method for detecting vocal cords in ET.

The parathyroid gland is generally pale orange‒red, while the adipose tissue is yellow. Therefore, they can easily be identified under endoscopic magnification [10]. However, in the process of clearing the central lymph nodes, the blood supply of the inferior parathyroid gland is easily affected or mistakenly cut [9, 10]. We also had similar experiences and believed that it was equally easy to identify parathyroid glands in patients who underwent the ETOVA or the ETAA. If the inferior parathyroid gland cannot be retained in situ, confirmation with a parathyroid hormone test and autotransplantation should be performed. As a result, no cases of hypoparathyroidism occurred in the ETOVA or ETAA group.

Infection has always been a concern in oral surgery because of the presence of clean and contaminated wounds, and it has been suggested that prophylactic use of antibiotics during the perioperative period and routine placement of drainage tubes after surgery could effectively prevent postoperative infection [13, 20, 27, 28]. In our experience, the mouth was rinsed with concentrated metronidazole after surgery, and ceftazidime (2 g) was prophylactically administered twice daily for 3 days. A drainage tube was routinely placed, and no infection occurred.

**Fig. 5:**
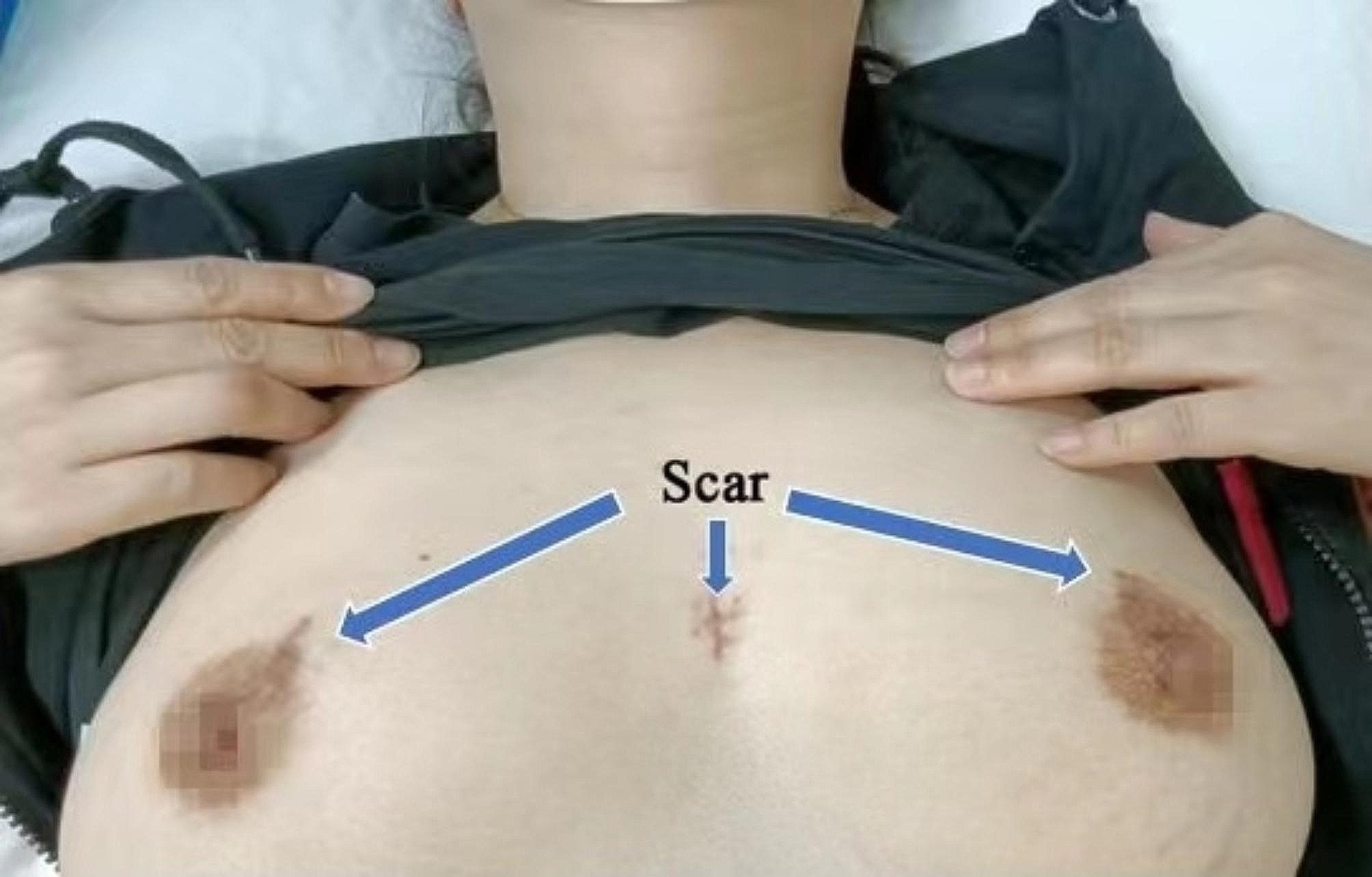
Scars after the ETAA

**Fig. 6:**
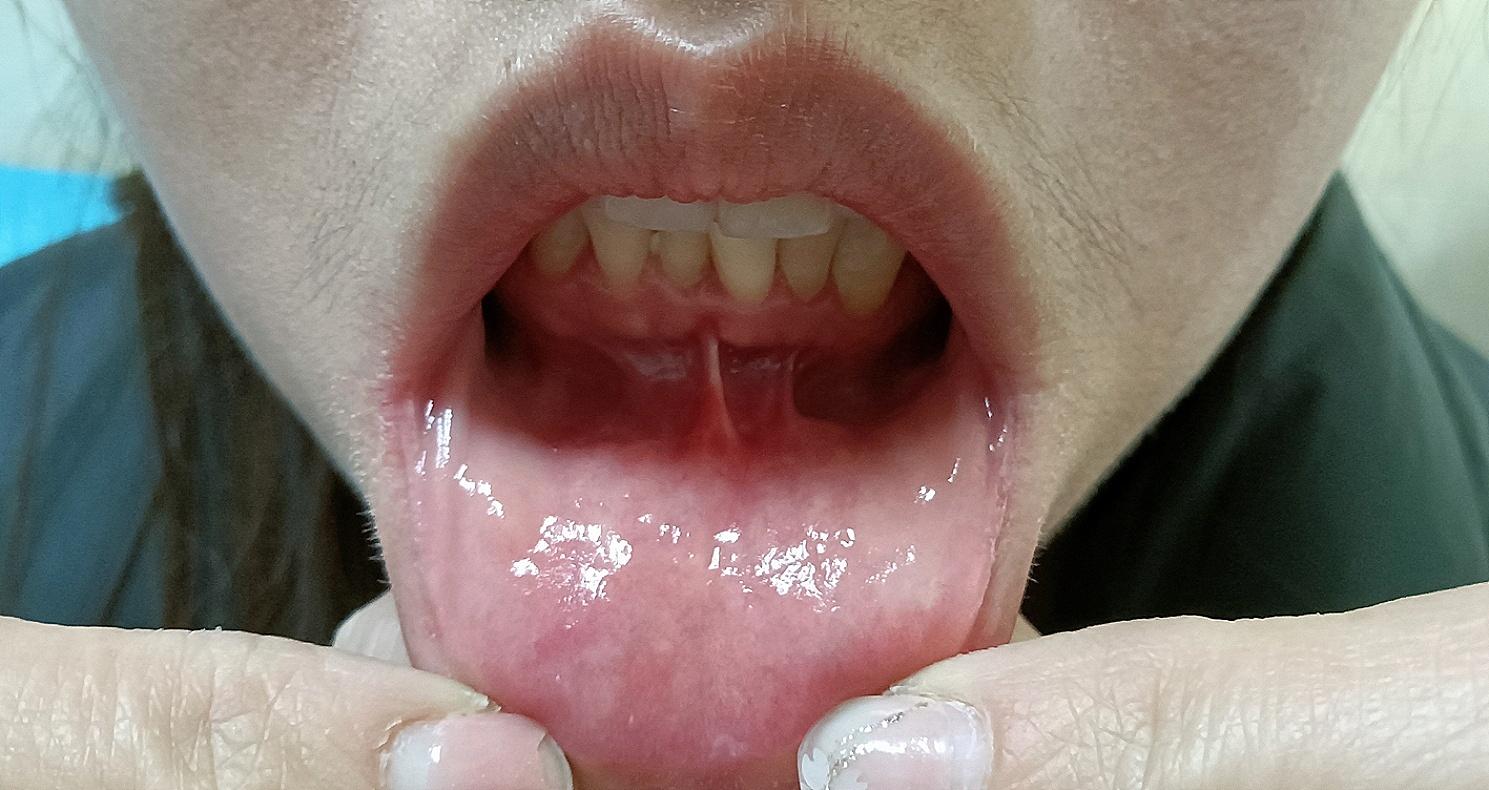
No scars after ETOVA

In the ETOVA, due to the close proximity of the oral cavity to the thyroid, the subcutaneous dissection tunnel is shorter, which thereby reduces the risk of surgical trauma [12]. Other endoscopic surgery methods, including the ETAA, require a larger extent of dissection to establish a workspace due to the greater distance between the operating trocar and the thyroid, resulting in more postoperative pain. Therefore, these procedures are not truly minimally invasive surgeries [12, 19, 27–29]. . Our results also showed that compared to patients in the ETAA group, patients in the ETOVA group had lower pain scores, and fewer patients reported neck discomfort. The greatest advantage of the ETOVA is that it is leaves no scars on the skin, while other endoscopic surgery methods, including the axillary breast approach, axillary approach, and areola approach, simply transfer skin incisions from the neck to the axillary fold and/or the breast and are not truly scar free (Fig. 5). Moreover, oral mucosal wounds healed without scarring after surgery (Fig. 6). Therefore, this method is more easily accepted by patients, especially young female patients [12, 27, 29]. As expected, our results showed that the cosmetic effect of the ETOVA was better than that of the ETAA.

Our results showed that one patient in the ETOVA group experienced tumor recurrence in the other thyroid lobe 21 months later, while another patient in the ETAA group experienced tumor recurrence in the other thyroid lobe 17 months later. There was no significant difference in the tumor recurrence rate between the two groups. Neither group had any occurrence of cervical lymph node or distant metastasis. Although the mean follow-up period was 50.45 ± 6.73 months in the ETOVA group and 52.60 ± 7.32 months in the ETAA group, the follow-up period was not long enough. Unlike other malignant tumors, PTC usually progresses more slowly and has a better prognosis, with a 10-year survival rate of over 90% [30]. Thus, it was difficult to compare the oncological outcomes of the two within the short follow-up period. However, randomized controlled trials with long-term follow-up assessments are needed to further evaluate oncological outcomes [11]. Recent studies have explored the possible prognostic value of single nucleotide polymorphisms (SNPs) of the vascular endothelial growth factor (VEGF) pathway in the prediction of recurrence of differentiated thyroid carcinoma (DTC). The CTG homozygous genotype of the VEGF pathway is believed to be closely related to the recurrence of nonadvanced DTC [31]. Liu also reported that the SNPs rs3024997 and rs3025040 in VEGFA were significantly associated with a higher risk of PTC [32]. Thus, the incorporation of VEGF SNP analysis in our future work could contribute to further investigations of the prognosis of PTC patients.

## Conclusions

We evaluated the feasibility, effectiveness and safety of the two endoscopic procedures. Our results showed that no significant difference existed between the two approaches in terms of surgical bleeding volume, postoperative drainage volume, length of hospital stay, or severity of postoperative complications. Due to the absence of visual limitations, the ETOVA is beneficial for clearing central lymph nodes. With a shorter anatomical channel, the ETOVA results in less postoperative pain and neck discomfort and has better cosmetic results. In summary, the ETOVA is not inferior to the ETAA in terms of the safety and curability of PTC and has advantages in central lymph node dissection, minimal invasiveness, and cosmetic results.

### Electronic supplementary material

Below is the link to the electronic supplementary material.

Supplementary Material 1

Supplementary Material 2

Supplementary Material 3

## Data Availability

All the data generated or analyzed during this study are included in the supplementary information files.

## Declarations

### Authors’ information

1 Department of Anesthesiology, Zhongshan Hospital of Xiamen University, Xiamen, China.

2 Department of General Surgery, Zhongshan Hospital of Xiamen University, Xiamen, China.

## Abbreviations

ETAA: Endoscopic Thyroidectomy Areola Approach
ETOVA: Endoscopic Thyroidectomy Oral Vestibular Approach
PTC: Papillary Thyroid Carcinoma
DTC: Differentiated Thyroid Carcinoma
OT: Open Thyroidectomy
ET: Endoscopic Thyroidectomy
RLN: Recurrent Laryngeal Nerve
SLN: Superior Laryngeal Nerve
PTH: Parathyroid Hormone
VAS: Visual Analog Scale
FNA: Fine Needle Aspiration
CO_2_: Carbon Dioxide
IONM: Intraoperative Neural Monitoring
