# Supplementary material for: Comparison of endoscopic thyroidectomy via the oral vestibule approach and the areola approach for papillary thyroid carcinoma
Source: BMC Surg. 2024 Apr 27;24:127. doi: 10.1186/s12893-024-02413-3 (PMC11055303; doi:10.1186/s12893-024-02413-3)
Supplement: Supplementary file 1 — Supplementary Material 1 [file 12893_2024_2413_MOESM1_ESM.doc]

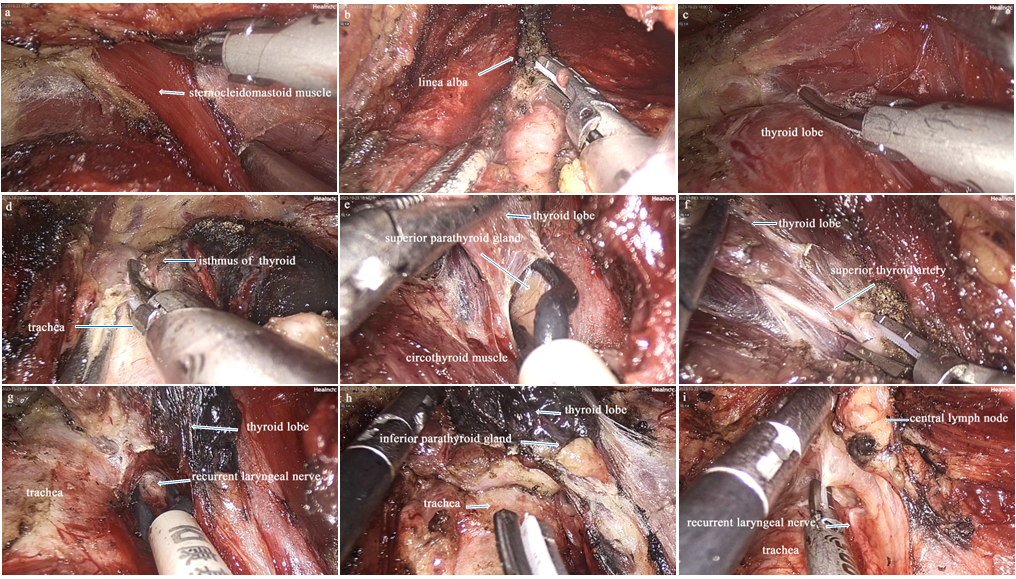


Supplementary Figure 1 Operative procedure of ETOVA. a Establishment of surgical space. b Separation of linea alba from the thyroid cartilage to the sternal notch. c Separation of thyroid surgical membrane. d Dissection of isthmus of the thyroid along the surface of the trachea. e Identification of the superior parathyroid gland. f Ligation of the superior thyroid artery on the thyroid capsule. g Exposure of recurrent laryngeal nerve along the trachea-esophageal groove. h Identification of the inferior parathyroid gland. i Dissection of central lymph node.


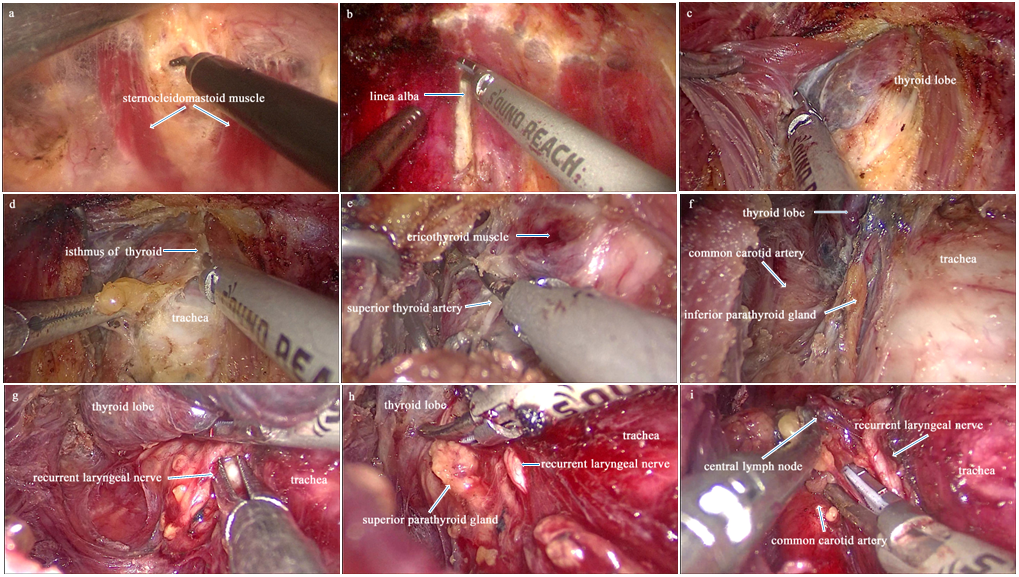


Supplementary Figure 2 Operative procedure of ETAA. a Establishment of surgical space. b Separation of linea alba from the sternal notch to the thyroid cartilage. c Separation of thyroid surgical membrane. d Dissection of isthmus of the thyroid along the surface of the trachea. e Ligation of the superior thyroid artery on the thyroid capsule. f Identification of the inferior parathyroid gland. g Exposure of recurrent laryngeal nerve along the trachea-esophageal groove. h Identification of the superior parathyroid gland. i Dissection of central lymph node.
