# Supplementary material for: Comparison of endoscopic thyroidectomy via the oral vestibule approach and the areola approach for papillary thyroid carcinoma
Source: BMC Surg. 2024 Apr 27;24:127. doi: 10.1186/s12893-024-02413-3 (PMC11055303; doi:10.1186/s12893-024-02413-3)
Supplement: Supplementary file 2 — Supplementary Material 2 [file 12893_2024_2413_MOESM2_ESM.pdf]

Code	Sex	Age(years)	Diameter of largest tummor (mm)	Capsule invasion	Extrathyroi
1	female	32	5	non	non
2	female	19	13	non	non
3	female	49	4	non	non
4	female	37	4	non	non
5	female	41	11	non	non
6	female	32	5	non	non
7	female	27	28	invasion	non
8	female	39	5	invasion	non
9	female	42	6	non	non
10	female	36	5	non	non
11	male	45	13	non	non
12	male	32	7	non	non
13	female	37	4	non	extra
14	male	40	9	non	non
15	female	36	5	non	non
16	male	38	19	non	non
17	male	33	15	non	non
18	female	62	8	non	non
19	female	40	6	non	non
20	female	50	7	invasion	non
21	female	31	5	non	non
22	female	25	20	non	non
23	male	31	9	non	non
24	female	30	2	non	non
25	female	28	14	non	non
26	female	46	8	non	extra
27	female	33	6	non	non
28	male	25	12	non	non
29	male	35	19	non	extra
30	female	45	3	non	non
31	female	36	9	non	non
32	female	37	9	non	non
33	female	39	6	non	non
34	female	32	13	invasion	non
35	female	31	6	non	extra
36	female	31	46	non	non
37	female	25	9	invasion	non
38	female	51	3	non	non
39	female	42	17	invasion	extra
40	male	36	4	non	extra
41	female	41	8	non	non
42	male	36	4	non	non
43	female	51	3	non	non
44	male	40	9	non	non
45	female	53	3	non	non
46	female	29	6	invasion	non
47	female	39	11	non	non
48	female	30	11	non	non
49	female	36	3	non	non
50	female	42	13	non	non

Location	LN (+)	Number of retrieved lymph nodes	TNM stage	OP time(min)
Right lobe	0	9	1	138
Right lobe	0	0	1	125
Right lobe	1	12	3	140
Right lobe	0	5	1	180
Right lobe	1	2	1	128
Right lobe	1	3	1	157
Right lobe	0	0	1	78
Right lobe	1	1	1	126
Right lobe	0	0	1	164
Left lobe	1	2	1	105
Right lobe	4	10	1	95
Left lobe	2	2	1	133
Right lobe	0	5	1	180
Left lobe	1	1	1	137
Left lobe	0	3	1	127
Right lobe	3	7	1	136
Left lobe	1	9	1	120
Left lobe	0	4	1	118
Left lobe	0	7	1	120
Right lobe	0	6	1	115
Left lobe	0	9	1	60
Left lobe	1	1	1	120
Left lobe	7	14	1	85
Left lobe	0	2	1	80
Right lobe	3	3	1	136
Left lobe	0	3	2	120
Right lobe	0	11	1	125
Right lobe	3	12	1	132
Right lobe	0	8	1	130
Left lobe	0	0	1	90
Right lobe	1	7	1	80
Left lobe	0	5	1	80
Left lobe	0	4	1	70
Right lobe	1	8	1	105
Right lobe	2	13	1	75
Right lobe	0	8	1	160
Right lobe	0	9	1	138
Left lobe	0	2	1	90
Right lobe	0	5	1	180
Left lobe	0	4	1	120
Right lobe	0	13	1	130
Left lobe	0	4	1	120
Left lobe	0	3	1	100
Left lobe	1	1	1	130
Left lobe	0	6	1	85
Bilateral lobe	2	11	1	200
Bilateral lobe	0	9	1	200
Bilateral lobe	0	4	1	188
Bilateral lobe	0	3	1	100
Bilateral lobe	11	26	1	90

Hospital stay(days)	Bleeding(ml)	Transient vocal cord palsy	Persistent vocal cord palsy
4	130		
3	65		
4	130		
4	225		
3	125		
6	210		
3	35		
2	100		
3	120		
4	175		
3	170		
4	160		
4	225	√	
5	205		
5	145		
4	155		
4	125		
5	105		
5	110		
4	90		
3	100		
4	90		
5	275		
4	80		
4	160		
5	230		
4	290		
3	110		
3	60		
6	100		
4	150		
3	105		
4	155		
3	125		
3	65		
3	100		
4	160		
4	115		
5	205		
3	130		
3	80		
3	130		
4	150		
4	205		
3	110		
5	240		√
5	215		
5	160		
6	210		
3	120		
		1	1

Superior laryngeal nerve injury	pre-PTH（ng/L）	post-PTH（ng/L）	pre-calcium(mm
	62	40.7	2.39
	54.4	35.2	2.45
	44	32.3	2.41
	42.1	29.8	2.31
	59.7	39.9	2.28
	76.8	59.5	2.34
	78.9	54.5	2.37
	41.4	27.8	2.53
	49.8	31.5	2.45
	48.9	39.6	2.27
	40.9	28.1	2.39
	42.3	24.9	2.44
	50.2	43.7	2.41
	44.8	35.7	2.33
	75.1	56.8	2.45
	43.9	38.6	2.45
	43.6	30.2	2.41
	64.1	38.6	2.28
	31.7	19.4	2.28
	60.9	44.9	2.56
	56	34.7	2.42
	45.3	37.4	2.42
	42.8	24.5	2.48
	44.1	27.5	2.36
	67.3	43.1	2.44
	37.3	22	2.26
	46.2	29.1	2.34
	22.5	18.5	2.57
	73.9	58.3	2.42
	37.5	24.4	2.51
	62.6	45.7	2.35
	65.5	46.4	2.42
	33.4	19.4	2.47
	52.4	38.6	2.43
	45	27.4	2.42
	45.5	23.7	2.43
	45.5	32.7	2.41
	76.5	54.9	2.21
	44.4	34.3	2.43
	59.9	43.6	2.33
	56.6	36.2	2.33
	35.8	22.9	2.4
	50.2	41.7	2.41
	69.7	54.6	2.41
	33.2	21.4	2.31
	50.1	36.3	2.32
	40.4	25.6	2.3
	33.8	19.3	2.43
	32.5	18.3	2.28
	42.9	26.3	2.38
0			

post-calcium(mmol/L)	Hypoparathyroidism	Postoperative subcutaneous fluid and would
2.16		
2.22		
2.33		
2.18		
2.15		
2.14		
2.2		
2.2		
2.28		
2.13		
2.2		
2.18		
2.23		
2.21		
2.34		
2.2		
2.32		
2.11		
2.2		
2.22		
2.23		
2.26		
2.2		
2.12		
2.31		
2.1		
2.18		
2.33		
2.21		
2.36		
2.24		
2.21		
2.16		
2.15		
2.11		
2.19		
2.28		
2.08		
2.22		
2.21		
2.17		
2.2		
2.21		
2.33		
2.2		
2.12		
2.11		
2.17		
2.1		
2.1		
	0	0

Postoperative pain（VAS 1）	Postoperative pain（VAS3）	Neck discomfort (POI
4.1	3.1	
4.1	3.8	
4.1	3.1	
5.1	4.1	√
4.4	3.4	
4.3	3.3	
3.9	2.9	
3.9	3.1	
3.8	2.8	
4.2	3.4	
4.4	3.4	
4.6	3.6	√
3.1	2.5	
4.1	3.3	
5.2	3.9	√
4.3	3.1	
5.2	3.9	√
4.1	3.1	
4.5	3.5	
4.1	3.1	
4.2	3.6	
4.3	3.7	
4.1	2.7	
4.2	3.5	
4.2	3.2	
4.7	3.7	√
4.1	2.8	
4	2,9	
4.2	3.2	
3.9	2.9	
3.9	2.7	
3.8	2.3	
4	2.5	
4	2.6	
4	3.4	
4.4	3.4	
4.5	3.5	√
4.3	2.7	
4.3	3.1	
3.9	2.9	
3.9	2.9	
4.5	3.5	√
5.2	4.2	√
4.4	3.4	
4.6	3.6	√
4.6	3.6	√
4.2	3.2	
4.7	4.1	√
4.9	3.9	√
5.1	3.4	√
		13

	Neck discomfort (POD 3 month)		Cosmesis	Recurrence	up period (r
			1		66
			2		66
			1		65
	√		1		65
			2		64
			1		63
			1		63
			1		60
			2		60
			1		52
			1		53
			1		54
			1		49
			2		48
	√		1		57
			2		57
	√		1		56
		1		56	
		1		56	
		2		56	
		1		55	
		2		55	
		1		55	
		1		54	
		1		54	
√		1		53	
		1		53	
			1		53
			2		52
			1		52
			1		52
			2		52
			2		52
			2	√	52
			1		52
			2		51
			2		51
			1		51
			1		41
			2		50
			1		49
			1		47
	√		2		40
			2		40
			1		45
			1		45
			1		43
	√		2		43
	√		1		36
			1		36
	7			1	
