# Supplementary material for: Comparison of endoscopic thyroidectomy via the oral vestibule approach and the areola approach for papillary thyroid carcinoma
Source: BMC Surg. 2024 Apr 27;24:127. doi: 10.1186/s12893-024-02413-3 (PMC11055303; doi:10.1186/s12893-024-02413-3)
Supplement: Supplementary file 3 — Supplementary Material 3 [file 12893_2024_2413_MOESM3_ESM.pdf]

Code	Sex	Age(years)	Diameter of largest tummor(mm)	Capsule invasion	l
1	female	51	6	non	
2	female	49	3	non	
3	female	23	7	non	
4	female	42	4	non	
5	female	57	8	invasion	
6	female	35	1	non	
7	female	39	8	non	
8	male	28	11	non	
9	female	38	2	non	
10	female	37	4	non	
11	female	42	12	non	
12	male	26	8	non	
13	female	29	8	non	
14	female	30	3	non	
15	female	45	8	non	
16	female	33	2	non	
17	female	30	6	non	
18	male	26	6	non	
19	male	29	4	non	
20	male	33	14	non	
21	female	32	9	non	
22	female	34	4	non	
23	female	48	8	non	
24	female	56	6	non	
25	male	38	3	non	
26	female	50	6	non	
27	male	50	4	non	
28	male	32	11	non	
29	female	31	17	invasion	
30	male	44	14	non	
31	male	39	6	non	
32	female	33	7	non	
33	female	35	6	non	
34	female	37	8	non	
35	female	42	6	non	
36	female	32	11	non	
37	female	50	7	non	
38	female	27	13	non	
39	female	43	11	non	
40	female	27	4	non	
41	female	29	5	non	
42	female	32	5	non	
43	male	26	6	non	
44	female	26	5	non	
45	female	36	12	non	
46	female	44	5	non	
47	female	30	9	non	
48	male	23	9	non	
49	female	42	12	invasion	
50	female	33	6	non	
51	female	28	4	non	
52	female	35	4	non	
53	female	26	7	non	
54	female	39	11	non	
55	female	30	9	invasion	
56	female	37	6	invasion	
57	female	38	3	non	

58	female	25	15	non
59	female	49	8	non
60	female	35	4	non
61	male	33	27	non
62	female	44	7	invasion
63	male	36	8	invasion
64	female	24	7	non
65	female	35	15	non
66	male	33	17	non
67	male	33	7	invasion
68	female	42	11	invasion
69	female	35	4	non
70	male	37	15	non
71	female	36	6	non
72	female	35	11	non
73	female	35	6	invasion
74	female	47	12	non
75	female	37	8	non
76	female	44	7	non
77	female	53	11	non
78	female	46	3	non
79	female	44	7	non

Extrathyroic	Location	LN (+)	retrieved lymph nodes	TNM stage	OP time(min)
non	Left lobe	0	2	I	114
non	Right lobe	0	0	I	121
non	Left lobe	3	6	I	190
non	Right lobe	0	25	I	250
extra	Right lobe	0	5	2	166
non	Left lobe	0	2	I	210
non	Right lobe	3	15	I	196
extra	Left lobe	2	6	I	173
non	Left lobe	0	3	I	190
non	Right lobe	1	3	I	225
non	Right lobe	0	13	I	190
non	Right lobe	0	2	I	150
non	Left lobe	0	8	I	210
non	Left lobe	0	7	I	154
non	Right lobe	2	5	I	195
non	Left lobe	0	10	I	112
non	Right lobe	0	3	I	128
non	Right lobe	10	15	I	170
non	Right lobe	0	4	I	157
non	Left lobe	1	5	I	180
non	Right lobe	1	2	I	200
non	Left lobe	0	6	I	134
non	Left lobe	0	2	I	115
non	Right lobe	3	22	2	134
non	Left lobe	0	5	I	140
non	Right lobe	0	9	I	172
non	Right lobe	0	6	I	180
non	Right lobe	7	15	I	190
non	Right lobe	1	3	I	193
non	Left lobe	1	11	I	150
non	Right lobe	0	5	I	189
non	Left lobe	0	3	I	118
non	Right lobe	0	3	I	155
non	Right lobe	0	5	I	155
non	Left lobe	0	6	I	110
non	Left lobe	0	7	I	138
extra	Right lobe	0	7	I	183
non	Left lobe	1	5	I	145
non	Left lobe	0	7	I	125
non	Left lobe	0	0	I	110
non	Left lobe	0	5	I	152
non	Left lobe	0	5	I	163
non	Right lobe	0	11	I	190
non	Left lobe	0	1	I	170
non	Left lobe	0	8	I	110
non	Right lobe	0	6	I	166
non	Left lobe	0	3	I	152
non	Right lobe	0	4	I	150
non	Right lobe	0	8	I	145
non	Right lobe	1	7	I	134
non	Right lobe	1	2	I	145
non	Left lobe	0	7	I	130
non	Left lobe	0	1	I	168
non	Left lobe	0	6	I	120
non	Right lobe	1	16	I	133
non	Right lobe	0	3	I	140
non	Left lobe	0	4	I	160

non	Right lobe	3	6	l	180
non	Right lobe	0	8	l	150
non	Right lobe	0	9	l	160
non	Right lobe	0	2	l	130
non	Right lobe	0	6	l	130
non	Left lobe	0	3	l	130
extra	Right lobe	0	5	l	120
non	Left lobe	0	8	l	120
extra	Left lobe	0	0	l	160
non	Left lobe	0	0	l	180
non	Right lobe	5	7	l	170
extra	Right lobe	2	3	l	120
extra	ilateral lobe	2	14	l	200
non	ilateral lobe	0	14	l	200
non	ilateral lobe	0	7	l	240
extra	ilateral lobe	1	11	l	220
non	ilateral lobe	6	11	3	125
extra	ilateral lobe	1	6	l	240
non	ilateral lobe	2	8	l	180
non	ilateral lobe	0	9	l	210
extra	ilateral lobe	4	24	3	240
non	ilateral lobe	2	22	l	190

Hospital stay(days)	Bleeding	Transient vocal cord palsy	Persistent vocal cor
4	130		
5	105		
4	230		
4	155		
4	20		
5	205		
4	75		√
3	100		
5	210		
4	105		
3	120		
4	140		
4	230		
5	245		
3	200		
3	55		
3	140		
4	130		
3	140		
4	210		
5	155		
4	200		
5	290		
4	250		
4	165		
3	150		
5	205		
5	235		
4	135		
4	170		
14	520		
4	160		
4	105		
5	130	√	
5	165		
4	115		
5	250		
3	130		
3	100		
3	105		√
3	130		
8	385		
4	220		
3	35		
4	185		
5	210		
5	235		
6	175		
3	125		
5	135		
5	100		
5	180		
3	135		
3	115		
3	195		
6	155		
5	195		

4	110		
3	130		
4	105		
4	150		
4	110		
3	70		
4	55		
5	100		
4	135		
4	195		
4	175		
5	285		
4	155		
6	205	√	
5	180		
5	155		
5	215		
6	355		
5	160		
6	185		
6	310		
4	170		
		2	2

Superior laryngeal nerve injury	pre-PTH（ng/L）	post-PTH（ng/L）
	70.4	45.7
	59.8	35.1
	38	22
	57.5	41.7
	32.3	18.2
	45.8	28.9
	39.7	28.2
	23.6	18
	52.2	32
	34.7	19.6
	51.7	40.3
	57.3	39.1
	28	16.5
	57.7	45.4
	57.4	36.5
	72.9	56.8
	72.8	49.3
	44.2	25.9
	76.2	74.7
	37	16.3
	33.7	20.1
	67.5	46.9
	52.6	39.4
	81.1	63.9
	58.1	48.9
	52.8	38.8
	45.2	36.5
	32.7	20.2
	37.4	24.1
	45.7	35.7
	53.3	40.9
	58	43.5
	52.9	38.2
	41.4	29.8
	28.3	17.2
	49.9	28.5
	45.9	30.1
	38.5	20.1
	45.1	31.7
	46.1	28.7
	55.7	41.2
	70.4	55.3
	42.9	28.4
	55.5	35.9
	67.3	45.3
	80.9	65.2
	46.6	30.4
	23.9	16.2
	56.6	32.8
	49.6	28.9
	51.3	46.8
	23.6	16.9
	66.4	42.7
	46	26.2
	50	32.5
	89.8	65.2
	60.9	43.6

	31.9	23.3
	56.7	39.9
	99.6	78.4
	56.3	39.2
	48.5	33.7
	41.7	26.7
	39.2	21.6
	62.7	55.1
	53.6	37.3
	53.1	34.2
	92.1	69.9
	56.8	40.4
	38.7	17.4
	20.6	17.6
	86	65.4
	61.4	36.3
	47.7	27.1
	82.9	62.9
	75.3	56.3
	31.7	19.8
	32.9	18
	46.6	30.1
0		

pre-calcium(mmol/L)	post-calcium(mmol/L)	Hypoparathyroidism	Postoperative subcut[illegible]
2.55	2.21		
2.35	2.19		
2.41	2.32		
2.38	2.14		
2.38	2.16		
2.27	2.09		
2.47	2.27		
2.59	2.31		
2.39	2.1		
2.5	2.25		
2.38	2.18		
2.45	2.23		
2.41	2.2		
2.35	2.3		
2.36	2.12		
2.42	2.26		
2.42	2.15		
2.44	2.23		
2.43	2.3		
2.47	2.02		
2.35	2.27		
2.45	2.33		
2.39	2.2		
2.45	2.2		
2.41	2.29		
2.34	2.21		
2.37	2.25		
2.57	2.28		
2.52	2.37		
2.43	2.32		
2.43	2.24		
2.44	2.33		
2.38	2.25		
2.38	2.16		
2.52	2.36		
2.38	2.15		
2.38	2.21		
2.31	2.23		
2.29	2.18		
2.38	2.24		
2.24	2.2		
2.37	2.19		
2.43	2.2		
2.32	2.17		
2.41	2.2		
2.35	2.2		
2.34	2.15		
2.51	2.22		
2.29	2.16		
2.26	2.07		
2.39	2.14		
2.37	2.33		
2.38	2.2		
2.43	2.17		
2.31	2.05		
2.3	2.2		
2.54	2.4		

2.38	2.27	
2.41	2.28	
2.32	2.2	
2.5	2.26	
2.36	2.21	
2.42	2.22	
2.31	2.17	
2.35	2.29	
2.47	2.23	
2.29	2.19	
2.54	2.31	
2.36	2.21	
2.53	2.2	
2.28	2.16	
2.4	2.2	
2.56	2.08	
2.31	2.07	
2.26	2.02	
2.38	2.15	
2.4	2.06	
2.47	2.1	
2.42	2.18	
		0

aneous fluid and would infection	Postoperative pain（VAS 1）	Postoperative pain（VAS 3）
	3.5	2.5
	3.8	2.8
	2.8	1.8
	3.6	1.6
	3.3	2.3
	3.8	1.8
	2.8	1.8
	2.8	1.8
	3.8	2.8
	3.5	2.5
	3.1	2.1
	3.3	2.3
	2.9	1.9
	3	2.6
	3.1	2.1
	2.9	1.9
	3.2	2.2
	3.3	1.9
	3.4	2.4
	3.5	2.8
	3.6	2.3
	3.7	2.9
	3.1	2
	3.2	2.2
	2.8	1.8
	3.1	2.5
	3.2	2.4
	4.1	2.9
	4.2	3.1
	4.3	3.1
	4.1	2.7
	3.9	2.9
	3.1	3.1
	3	2.2
	3.2	2.3
	3.6	2.6
	2.8	2
	3.4	2.4
	3.1	2.4
	2.9	1.9
	2.9	2.3
	3.2	2.2
	3.2	2.5
	3.3	2.3
	3.4	2.4
	3.4	2.6
	3.2	2.2
	2.8	2.2
	3	2.3
	3	2.3
	2.9	1.9
	2.9	1.9
	3	3
	3	2.2
	3	2.5
	4	2.4
	2.8	2.8

	3	2
	3	2.1
	2.8	1.9
	2.8	2.1
	3	2.3
	3	2.3
	2.9	2.1
	3	2.3
	3.1	2.1
	4.3	3.3
	3.2	2.2
	3.2	2.2
	3.3	2.9
	3.1	2.1
	3.2	2.2
	3.4	2.4
	3.6	2.6
	3.8	2.8
	3.9	1.9
	4	2.8
	4.1	3.1
	4.2	2.9
0		

Neck discomfort (POD 1 month)	Neck discomfort (POD 3 month)	Cosmesis	Recurrence	up period (ı
		1		64
		1		64
		1		62
		1		62
		1		62
		1		60
		1		60
		1		59
		2		59
		1		58
		1		58
		1		58
		1		58
		1		58
		2		58
		1		57
		1		57
		2		56
		1		56
		1		56
		1		55
		1		55
		1		54
		1		54
		1		54
		1		54
		1		53
√		1		53
√		1		52
√	√	1		52
√		2		52
		1		50
		1		50
		1		50
		1		50
		1		50
		1		48
		2		48
		1		47
		1		47
		1		46
		1		46
		1		44
		1		44
		1		42
		1		42
		1		46
		1		48
		1		50
		1		44
		1	√	49
		1		49
		1		38
		1		38
		1		38
		1		47
		2		47

		1		46
		1		52
		1		52
		1		47
		1		47
		1		40
		1		42
		1		44
		1		46
√		1		36
		1		36
		1		46
		1		46
		1		45
		1		44
		1		46
		1		45
		1		44
		1		53
		1		54
√	√	1		54
√		1		53
7	2		1	
